# Graph-Based Framework with Waveform-Informed Connectivity for Multi-Label Partial Discharge Source-Type Classification

**DOI:** 10.3390/s26123903

**Published:** 2026-06-19

**Authors:** Leandro José Duarte, Andréia Coelho Domingos, Alan Petrônio Pinheiro, Lorenço Santos Vasconcelos, Fabrício Augusto Matheus Moura, Fernando Elias de Freitas Fadel, Patrícia Naomi Sakai

**Affiliations:** 1 Smart Grids Laboratory, Faculty of Electrical Engineering, Federal University of Uberlândia, Uberlândia 38408-100, MG, Brazil; andreia.domingos@ufu.br (A.C.D.); alanpetronio@ufu.br (A.P.P.); lorenco.santos@ufu.br (L.S.V.); fabricio.moura@ufu.br (F.A.M.M.); 2 Petróleo Brasileiro S.A. (Petrobras), Rio de Janeiro 20231-030, RJ, Brazil; fernandofadel@petrobras.com.br (F.E.d.F.F.); patricia.sakai@petrobras.com.br (P.N.S.)

**Keywords:** edge-conditioned convolution, graph neural networks, multi-label classification, partial discharge source-type classification, phase-resolved partial discharge

## Abstract

Partial discharge (PD) source-type classification is essential for condition-based maintenance of high-voltage apparatus. Existing approaches based on grid discretizations of phase-resolved partial discharge (PRPD) patterns suffer from performance degradation under stochastic interference and multi-source conditions. This paper proposes a graph-based framework that integrates the morphological characterization of raw high-frequency PD waveforms with the phase-amplitude position of individual discharge events to enable multi-label classification, identifying multiple PD sources coexisting within a single test. The framework operates through three stages: a multi-task neural network extracts per-pulse embeddings and confidence scores; a construction procedure establishes selective graph connectivity based on spatial proximity and morphological similarity; and an edge-conditioned graph neural network performs classification via message passing weighted by multimodal edge attributes. Experimental evaluation on PD measurements acquired in accordance with IEC 60270 shows that the proposed framework achieves a Matthews correlation coefficient (MCC) of 0.98 and an exact match ratio of 0.97 across single-source, noisy, and multi-source conditions, substantially outperforming histogram- and set-based baselines. The framework maintains an MCC of 0.97 in multi-source scenarios, where its advantage over existing methods is most pronounced. Cross-domain evaluation on an independent dataset acquired with different laboratory equipment confirms the approach’s robustness, achieving an MCC of 0.93 without retraining. Finally, an ablation study demonstrates that the joint removal of morphological similarity filtering and confidence-based node filtering and edge gating reduces the MCC by 0.25, confirming the critical role of the waveform-informed relational structure.

## 1. Introduction

Partial discharge (PD) is a primary indicator of insulation degradation in high-voltage apparatus. Its occurrence reflects a progressive deterioration of the dielectric system, which can lead to operational risks and to the failure of critical assets such as power transformers, gas-insulated switchgear (GIS), and insulated power cables [1,2,3]. In PD monitoring, the classification of PD source types—including corona, internal, and surface—constitutes a fundamental diagnostic task, as each source type is associated with a different severity level and determines specific corrective actions for maintenance planning [2,3,4,5].

Within this context, phase-resolved partial discharge (PRPD) patterns represent one of the standard formats used by both domain experts and automated systems for PD source-type classification [4,6,7]. By mapping pulses relative to the phase of the applied alternating current (AC) voltage, these patterns provide a statistical profile characteristic of the physical nature of the defect, since the discharge mechanism governs where each source type appears along the voltage cycle; for instance, charge-trapping effects position internal discharges differently from surface discharges [4,6,8,9]. While manual characterization has traditionally been employed, the expansion of smart grids and condition-based maintenance (CBM) generates data volumes that necessitate robust automated classification systems [2,4,10,11,12,13,14].

Early automated approaches for PD source-type classification used traditional machine learning algorithms that relied on manual feature engineering [4,8,15,16]. In these methods, the selection of statistical descriptors determined the capacity of the model to distinguish between different PD patterns. More recently, the field has transitioned toward deep learning architectures, particularly convolutional neural networks (CNNs), to automate the classification task [7,17,18,19,20]. By treating PRPD data as static grid-based representations such as 2D intensity maps or images, these networks learn spatial patterns directly from the data [17,21,22,23,24]. Although image-based models achieve high performance on controlled benchmarks, diagnostic performance degrades under stochastic interference, particularly when multiple PD source types coexist within the same PD test [4,8,25]. A recent review further observes that models operating on such representations tend to learn superficial statistical regularities rather than the physical signatures of each discharge type, sustaining a high misclassification rate between mechanisms with similar patterns [26].

This degradation stems from the limitations of grid-based representations under noisy and multi-source conditions. In these scenarios, stochastic interference overlaps with PD patterns within the phase-amplitude domain, inducing binning artifacts where noise components are misinterpreted as discharge activity. Furthermore, grid-based discretizations are poorly suited to the sparse and non-uniform point-cloud nature of PD activity in such environments, either introducing artifacts in void regions or losing resolution in high-density clusters [24,27]. More fundamentally, the discriminative information for source-type classification lies in the pairwise relationships among events—multiple discharges from the same source share characteristic waveform signatures—rather than in the aggregate distribution of activity within phase-amplitude regions. Grid-based representations dissolve these pairwise relationships by aggregating events into fixed bins, retaining only the marginal occupancy of each bin and discarding the relational structure that distinguishes coherent discharge from stochastic interference [28].

To address these limitations, this work proposes a graph-based framework for PD source-type classification that combines morphological characterization of the raw high-frequency PD waveforms with the conventional phase-amplitude representation provided by PRPD patterns. This integration supplies the graph construction with waveform-level information absent from standard PRPD images, yielding a graph in which nodes correspond to individual discharge events and edges reflect both spatial proximity and morphological similarity. Unlike grid-based approaches that operate on a fixed image, the proposed framework derives both graph connectivity and message passing directly from the individual waveforms. Through this selective connectivity, it preserves the intrinsic relationships between pulses and attenuates noise components that lack structural consistency with valid discharge events, even in complex scenarios where distinct discharge sources overlap.

The primary contributions of this work are as follows:

- A multi-task neural network for morphological characterization of high-frequency PD waveforms that jointly extracts discriminative embeddings and per-pulse confidence scores.
- A graph construction procedure that integrates waveform morphology and confidence information to establish selective connectivity between discharge events, thereby enabling structural filtering of stochastic interference.
- An edge-conditioned graph neural network for multi-label classification of PD source types (corona, internal, and surface) that conditions message passing on multimodal edge attributes encoding geometric proximity, morphological similarity, and per-pulse confidence.

To demonstrate the practical robustness of these methodological advances, the framework is rigorously validated on an independent cross-domain PD dataset acquired with different laboratory equipment and test-object geometries, achieving high generalization without any retraining or fine-tuning.

The remainder of this work is organized as follows: Section 2 reviews related work on PD source-type classification; Section 3 details the proposed graph-based framework, including the morphological characterization stage, the graph construction procedure, and the edge-conditioned classification stage; Section 4 describes the datasets, the data augmentation procedure, the training configuration, and the baseline methods; and Section 5 presents the experimental results, including cross-domain evaluation on an external dataset, an ablation and sensitivity analysis, and a computational characterization of the framework, followed by the conclusions in Section 6.

## 2. Related Work

PD source-type classification has progressed from manual analysis toward automated frameworks [1,4,10]. Maintaining classification reliability against stochastic interference and simultaneous PD source types—a prerequisite for modern CBM—remains a primary challenge [2,3,5,8,11,12]. This section reviews the progression from statistical feature extraction and grid-based deep learning toward graph-based representations, identifying the limitations that motivate the proposed framework.

### 2.1. Statistical Feature Extraction and Conventional Machine Learning

Early research in automated PD source-type classification focused on quantifying PD activity through statistical and temporal descriptors [4,6,8]. These classical pipelines typically model the PRPD distribution using statistical moments such as skewness, kurtosis, and asymmetry to characterize PD patterns, and can also employ time–frequency transformations such as the discrete wavelet transform to capture pulse-level dynamics [4,15,16,26]. These descriptors form the input for the conventional machine learning classifiers—including support vector machines (SVMs), random forests, and *k*-nearest neighbors—which established the initial benchmarks for PD source-type classification [8,16,25].

However, the feature engineering process constrains the discriminative capacity of these models. Handcrafted features do not adequately account for the nonlinear variations induced by signal propagation attenuation and stochastic interference [4,17,29]. Moreover, these static descriptors lack the sensitivity required to separate genuine discharge activity from such interference. Because these methods collapse the high-dimensional morphological signatures of individual waveforms into aggregated metrics, their applicability in high-interference scenarios is limited, which remains a major obstacle for reliable maintenance planning [8,24,30].

### 2.2. Grid-Based Deep Learning

To mitigate dependence on manual feature engineering, deep learning has been adopted as a common paradigm for PD source-type classification. By using CNNs, these approaches enable automated learning of hierarchical spatial patterns directly from data [17,18,19]. Prevalent methodologies project event-based PD data into 2D accumulated matrices, imposing a static image representation that assumes spatial stationarity in the PD pattern [7,21,22,23].

However, grid-based representations impose assumptions inherited from convolutional architectures that are not satisfied by PD data. CNNs presuppose a dense intensity field with local statistical dependencies, which allows convolutional filters to extract patterns by aggregating information from spatially adjacent pixels [24,27]. By contrast, PD activity is a sparse point cloud of discrete discharge events, each individually characterized by a high-frequency waveform; the dependencies between events arise from physical discharge mechanisms—two pulses originating from the same defect exhibit similar morphological signatures regardless of their location in the phase-amplitude plane—rather than from pixel adjacency. Therefore, the discretization step required to fit PD data onto a fixed pixel grid eliminates the per-event waveform identity that distinguishes valid pulses from stochastic interference and separates one PD source type from another, retaining only the aggregate bin statistics that the convolutional architecture is designed to process.

The consequences of this mismatch become pronounced under stochastic interference and multi-source conditions. In the phase-amplitude domain, such interference overlaps with discharge activity and the binning operation merges valid pulses with it, eliminating discrimination at the bin level [4,8,30]. When multiple PD source types coexist within the same PD test, overlapping contributions from distinct sources are further collapsed into shared bins, erasing the distributional signatures required for reliable separation [4,29].

These limitations are not incidental to a specific CNN architecture but follow fundamentally from the inductive bias of the grid representation itself. Casting a PD test as a dense phase-amplitude image imposes a fixed Euclidean topology in which connectivity is predetermined by pixel adjacency and the convolutional kernels assume locality and spatial stationarity; therefore, the representation encodes the per-bin pulse density and treats all events falling within a bin as interchangeable. Two consequences follow directly: discharges from distinct sources that occupy the same phase-amplitude region are merged into shared bins and can no longer be separated, and the representation is tied to the absolute pulse density on which the network was calibrated, meaning that a shift in density degrades it even when the underlying discharge morphology is unchanged. A graph built over individual discharge events embodies a different inductive bias. Connectivity is not fixed a priori but inferred from the data, combining phase-amplitude proximity with waveform-morphology similarity so that message passing aggregates over morphologically coherent neighborhoods rather than over geometric bins. This relational structure separates co-located sources whenever their high-frequency signatures differ, and adapts to the number of available events (a sparse PD test yields a smaller but valid graph) rather than presupposing a calibrated bin occupancy. The empirical consequences of this distinction are isolated in Section 5 through event-level set-based controls together with a cross-domain sparsity analysis.

### 2.3. Graph-Based Representations for PD Analysis

Recent work has explored graph-based representations to address structural limitations of grid-based approaches in PD diagnostics. Graphs offer a representation that is aligned with point-cloud inputs: they are permutation-invariant with respect to the constituent events and can accommodate relational structure that need not follow spatial adjacency. These efforts span different tasks and granularities; Zhang et al. [31] constructed graph nodes from subregions of a discretized PRPD matrix for semi-supervised fault recognition in transformers, Roy et al. [32] applied image visibility graph theory to PRPD plots as a feature extraction step for defect identification in cross-linked polyethylene (XLPE) cables, and Zhang et al. [28] built graph adjacency matrices from signal-level feature similarity for binary PD detection in overhead lines. These approaches demonstrate the potential of graph-based representations in the PD domain. However, in each case the graph structure is derived either from the discretized PRPD image or from features of complete measurement signals, with nodes representing image patches or entire PD tests rather than individual discharge events. As a result, the morphological characteristics of individual high-frequency PD waveforms are not incorporated into the graph construction, and the topological relationships between pulses within a single PD test remain unexploited. The framework proposed in this work operates at the level of individual discharge events, using per-pulse morphological embeddings and confidence scores to determine graph connectivity. In this formulation, each discharge event is a node with its own waveform-derived identity; edges carry multimodal attributes encoding the spatial proximity in the continuous phase-amplitude plane, morphological similarity between waveforms, and gating reliability of the connected events. This contrasts with the static pixel adjacency imposed by a discretized image grid and aligns the inductive bias of the representation with the point-cloud structure of PD activity, an advantage that the comparative evaluation in Section 5.2 confirms is most pronounced under multi-source conditions.

## 3. Proposed Graph-Based Framework

**Figure 1:**
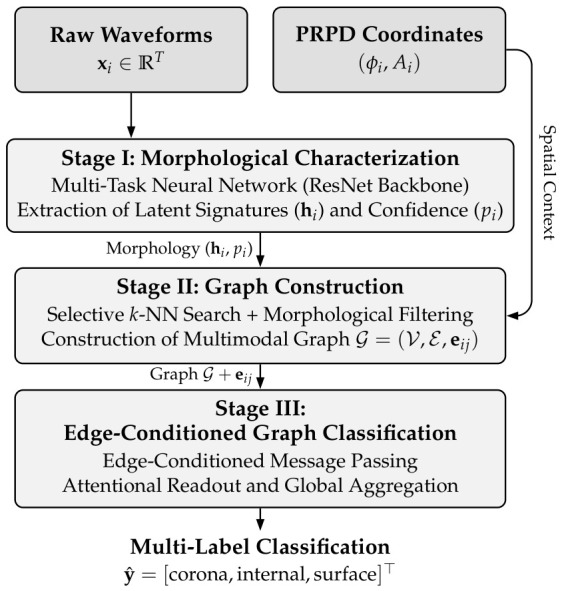
Overview of the proposed graph-based framework for PD source-type classification. The framework integrates morphological analysis of high-frequency PD waveforms with PRPD patterns to construct graphs that selectively retain reliable discharge events.

The proposed framework, illustrated in Figure 1, consists of three main stages: (i) a multi-task neural network that processes raw high-frequency PD waveforms to extract discriminative morphological embeddings and per-pulse confidence scores; (ii) a graph construction stage that builds a graph representation using both phase-amplitude coordinates and waveform features; and (iii) an edge-conditioned graph neural network that performs message passing over the constructed graphs to conduct multi-label classification of PD source types that may occur simultaneously.

### 3.1. Problem Formulation

A PD test is represented by a set of measurements $S={\left\{\left(\phi_{i},A_{i},x_{i}\right)\right\}}_{i=1}^{N}$, where *N* denotes the number of detected events, each characterized by a phase angle $\phi_{i}\in \left[0,2\pi \right)$, peak amplitude $A_{i}\in R$, and high-frequency waveform $x_{i}\in R^{T}$, with *T* denoting the temporal dimensionality of the signal. The objective is to learn a mapping function $\Phi :S\to {\left\{0,1\right\}}^{3}$ such that $\hat{y}=\Phi \left(S\right)$, where $\hat{y}$ is a binary vector indicating the presence or absence of each of the three PD source types (corona, internal, and surface). Multiple types may occur simultaneously within the same PD test.

### 3.2. Stage I: Morphological Characterization

**Figure 2:**
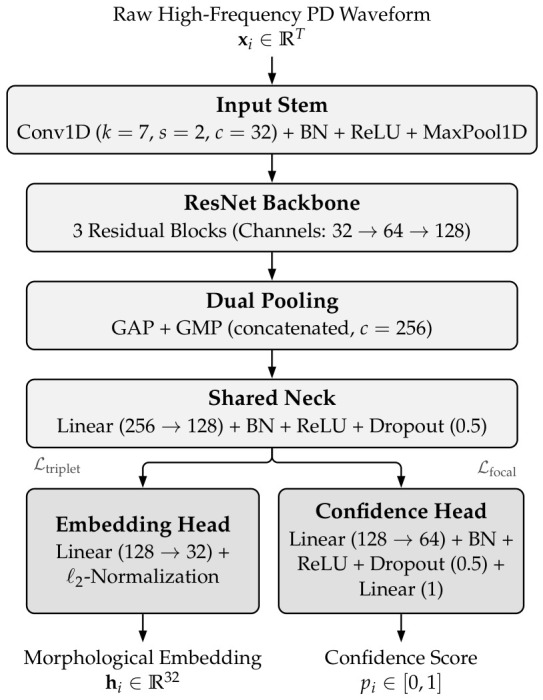
Architecture of the multi-task morphological characterization stage. The shared ResNet backbone extracts hierarchical features from raw waveforms and directs them to specialized heads for embedding generation and confidence estimation.

The phase-amplitude domain alone provides limited discriminative information for isolating valid PD pulses from stochastic interference. To address this limitation, the proposed framework incorporates morphological analysis of the raw high-frequency PD waveforms. This stage employs a multi-task neural network with a one-dimensional residual network (ResNet) backbone as a shared feature extractor that branches into two specialized heads. The overall architecture of this stage is illustrated in Figure 2, while the specific layer configurations and hyperparameters are detailed in Table 1.

The backbone depth, pooling strategy, and embedding dimensionality *d* all follow from the morphological structure of the input. The choice of three residual blocks balances representational depth against overfitting risk on the morphological dataset $D_{M}$, which contains 3650 PD waveforms from 73 physical sources together with 6000 noise samples. Shallower configurations limit the hierarchical composition of features required to capture transient discharge morphology, while deeper configurations introduce capacity that the available training data do not support. The dual-pooling strategy concatenates global average pooling and global max pooling to preserve two complementary descriptors of each waveform—the average pooling captures the envelope of the transient signal, while the max pooling captures its peak amplitude—whereas single-pooling configurations discard one of these descriptors and reduce the separability of source types in the latent space. The embedding dimensionality is set to d=32, since lower values reduce the separation between sources and higher values introduce redundancy without proportional gain. These choices are corroborated by the architectural sensitivity analysis reported in Section 5.5.

The two heads operate as follows:

- Embedding head: This head maps the waveform $x_{i}$ into a latent vector $h_{i}\in R^{32}$ within a normalized metric space. The head is trained with triplet loss such that pulses from the same physical source exhibit high cosine similarity while pulses from different sources are separated.
- Confidence head: This head estimates a scalar $p_{i}\in \left[0,1\right]$, representing the probability that the *i*-th event corresponds to a valid PD pulse rather than stochastic interference. This head is trained using the focal loss.

The network is trained one time, then its parameters are frozen for the remainder of the pipeline.

### 3.3. Stage II: Graph Construction

This stage converts the set of discharge events into a graph representation, using the morphological embeddings $h_{i}$ and confidence scores $p_{i}$ generated by the multi-task network in Stage I. A confidence threshold $\tau_{p}$ is applied first; any event with $p_{i}<\tau_{p}$ is discarded (hard node filtering). PD tests that retain fewer than a predefined minimum number of events after this filtering are also excluded. The remaining events constitute the nodes of the graph. Thus, node retention is the result of two successive selection stages: the upstream event-detection threshold (the acquisition peak detector for the in-domain data and the 6σ amplitude criterion for the cross-domain data), which fixes the set of *N* candidate events; and the confidence threshold $\tau_{p}$ applied here, which retains as nodes only those candidates that the morphological network identifies as valid discharges. The detection threshold determines how many candidates enter the pipeline, while the confidence gate determines which of them become graph nodes; thus, the node set is shaped primarily by the morphological gate rather than by the detection threshold alone.

Each node $n_{i}\in V$ is represented by the feature vector

(1) $$u_{i}={\left[{\tilde{\phi }}_{i},{\tilde{A}}_{i},p_{i}\right]}^{⊤},$$

where ${\tilde{\phi }}_{i}=\phi_{i}/2\pi$ is the normalized phase angle and ${\tilde{A}}_{i}$ is the normalized peak amplitude.

Graph connectivity is constructed via selective *k*-nearest neighbor search performed exclusively in the normalized phase-amplitude space $\left({\tilde{\phi }}_{i},{\tilde{A}}_{i}\right)$. For each node, spatial candidate neighbors are identified first; an edge is retained only if the morphological similarity weight $W_{\mathrm{morph}}\left(i,j\right)$ between the corresponding embeddings satisfies $W_{\mathrm{morph}}\left(i,j\right)\geq \tau_{\mathrm{morph}}$, where $\tau_{\mathrm{morph}}$ is a minimum morphological similarity threshold. This criterion connects only pulses with consistent high-frequency signatures; the same weight, defined below, subsequently modulates the importance of each retained edge during message passing.

Each retained edge (i,j) carries a three-dimensional attribute vector that conditions message-passing in the classification stage:

(2) $$e_{ij}=\left[W_{\mathrm{geo}}\left(i,j\right),\;W_{\mathrm{morph}}\left(i,j\right),\;W_{\mathrm{gate}}\left(i,j\right)\right].$$

The three components are defined as follows:

- Geometric proximity ($W_{\mathrm{geo}}$): This weight quantifies local connectivity through a self-tuning Gaussian kernel:
  (3) $$W_{\mathrm{geo}}\left(i,j\right)=\exp\left(-\frac{∥{\tilde{q}}_{i}-{\tilde{q}}_{j}{∥}_{2}^{2}}{2\sigma_{ij}^{2}}\right),$$
  where ${\tilde{q}}_{i}={\left[{\tilde{\phi }}_{i},{\tilde{A}}_{i}\right]}^{⊤}$ denotes the normalized phase-amplitude coordinates, $\sigma_{ij}^{2}={\left(\left(\delta_{i}+\delta_{j}\right)/2\right)}^{2}+\epsilon_{0}$, with $\epsilon_{0}$ a small constant ensuring numerical stability, and $\delta_{i}$ denotes the distance to the *k*-th valid neighbor of node $n_{i}$.
- Morphological similarity ($W_{\mathrm{morph}}$): This weight quantifies the similarity between the latent signatures h extracted in Stage I:
  (4) $$W_{\mathrm{morph}}\left(i,j\right)={\left(\frac{1+\cos(h_{i},h_{j})}{2}\right)}^{\gamma },$$
  where γ controls the sensitivity of the morphological weighting.
- Gating reliability ($W_{\mathrm{gate}}$): This weight quantifies the joint reliability of the connected nodes from the confidence scores:
  (5) $$W_{\mathrm{gate}}\left(i,j\right)=\frac{p_{i}+p_{j}}{2}.$$

The per-test time complexity of the graph construction is O(N+|V|log|V|), where *N* is the number of detected events and |V|≤N is the number of nodes retained after confidence gating. This bound follows from four operations: (i) hard node filtering, which thresholds the confidence score of each of the *N* detected events and is O(N); (ii) construction of a spatial index over the normalized phase-amplitude coordinates of the |V| retained nodes, which is O(|V|log|V|); (iii) the *k*-nearest neighbor search performed against this index in the two-dimensional space $\left({\tilde{\phi }}_{i},{\tilde{A}}_{i}\right)$, which is also O(|V|log|V|); and (iv) morphological edge filtering, which evaluates a cosine similarity only for the *k* candidate neighbors retrieved per node over the fixed *d*-dimensional embeddings, contributing O(kd|V|). As *k* and *d* are constants of the framework configuration, the dominant terms are those of node filtering and the spatial search; this construction avoids the quadratic cost $O(N^{2})$ of dense pairwise comparison strategies. Because the confidence gating discards the large fraction of events produced by background interference, |V| is typically well below *N* and the resulting edge set is sparse. Each PD test is processed independently, so the procedure scales linearly in the number of tests in a stream; combined with the batch-level parallelism of the spatial search, the cost of graph construction as measured in practice grows sublinearly with *N* over the evaluated range, as reported in Section 5.6.

### 3.4. Stage III: Edge-Conditioned Graph Classification

**Figure 3:**
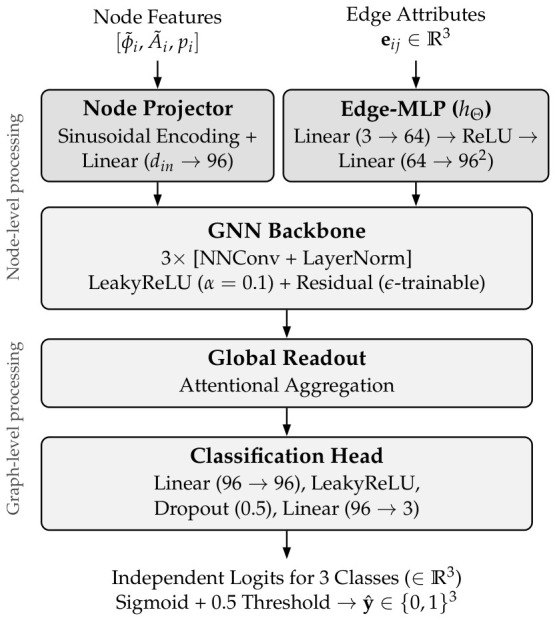
Architecture of the edge-conditioned graph classifier. The network processes node features and multimodal edge attributes through edge-conditioned convolutions followed by attentional aggregation for graph-level classification.

The final stage of the framework performs multi-label classification of PD source types by processing the constructed graph G through an edge-conditioned graph neural network (GNN). As illustrated in Figure 3, the network processes node-level features and multimodal edge attributes. By conditioning the convolutional filters on $e_{ij}$, the architecture enables a dynamic message-passing scheme that weights information exchange according to geometric proximity, morphological similarity, and gating reliability. The specific layer configurations and hyperparameters are detailed in Table 2.

#### 3.4.1. Edge-Conditioned Message Passing

The architecture updates the latent representation of each PD event through a recursive message-passing scheme. The initial node features $\left[{\tilde{\phi }}_{i},{\tilde{A}}_{i},p_{i}\right]$ are augmented with sinusoidal positional encodings (yielding an intermediate input dimensionality $d_{in}$) before being projected into a 96-dimensional latent space. In each layer *l*, the embedding $z_{i}^{\left(l\right)}$ for a node $n_{i}\in V$ is updated as follows:

(6) $$\begin{array}{c} z_{i}^{\left(l\right)}=\mathrm{LeakyReLU}(\mathrm{LayerNorm}(\left(1+\epsilon^{\left(l\right)}\right)z_{i}^{\left(l-1\right)}+\\ \sum_{j\in N(i)}h_{\Theta }^{\left(l\right)}\left(e_{ij}\right)z_{j}^{\left(l-1\right)})) \end{array}$$

where $h_{\Theta }^{\left(l\right)}$ denotes the Edge-MLP that maps the multimodal edge attributes $e_{ij}\in R^{3}$ to the space of the convolutional weights. This mechanism conditions message passing on the structural relationships within the graph.

#### 3.4.2. Global Readout and Classification Output

To derive a graph-level descriptor $z_{G}$, an attentional aggregation mechanism is employed. This mechanism computes learnable importance coefficients for each node, allowing the network to focus on the morphological signatures most characteristic of the underlying PD source types while attenuating the influence of stochastic interference. The global embedding $z_{G}$ is mapped to the output space through a classification head comprising two linear layers with LeakyReLU activation and dropout, producing independent logits for each of the three PD source types. During inference, the probabilities for each PD source type are obtained via the sigmoid function, and a threshold of 0.5 is applied to produce the final multi-label prediction.

### 3.5. Training Procedure

The optimization of the framework proceeds through three sequential stages; only Stage I and Stage III involve training, while Stage II consists solely of inference with the frozen encoder from Stage I.

Stage I trains the multi-task network on individual waveforms, optimizing the embedding head with triplet loss and the confidence head with focal loss; its parameters are then frozen for the remainder of the pipeline. Stage II applies this frozen encoder to each PD test, maps the high-frequency waveforms to embeddings and confidence scores, discards events below the confidence threshold, excludes PD tests with fewer than the minimum number of retained events, and constructs the graph with selective connectivity. Stage III trains the edge-conditioned graph neural network on the resulting graphs, optimizing a binary cross-entropy objective suited to the multi-label setting. The optimization hyperparameters for Stages I and III are reported in Section 4.2.

## 4. Experimental Setup

This section describes the experimental setup used to evaluate the proposed graph-based framework for PD source-type classification. It details the datasets, the data augmentation procedure, the training configuration, and the baseline methods employed for benchmarking. All methods share the same stratified 80/10/10 partition for training, validation, and testing, which divides the topological dataset $D_{T}$ into 720 training, 90 validation, and 90 test PD tests; the morphological dataset $D_{M}$ follows the same 80/10/10 proportion, stratified at the level of physical sources.

### 4.1. Datasets

The evaluation of the proposed framework uses two independent data sources. The first comprises PD measurements provided by the Smart Grids Laboratory (LRI), which are used for training, validation, and testing. The second is a publicly available dataset of controlled PD measurements [33], which is used exclusively for cross-domain evaluation. All LRI data were acquired in accordance with IEC 60270 [34] recommendations for PD measurement on high-voltage insulated cables. Each detected event comprises a high-frequency waveform $x_{i}$, its phase angle $\phi_{i}$, and its peak amplitude $A_{i}$. The waveforms consist of 500 samples, corresponding to a 5 μs temporal window captured at a sampling rate of 100 MSa/s.

#### 4.1.1. Data Organization

The PD measurements from LRI were organized into two independent datasets to support the modular training procedure described in Section 3.5:

- Morphological Dataset ($D_{M}$): Contains 3650 PD waveforms from 73 distinct physical sources and 6000 noise samples. This dataset is used to train the multi-task neural network for morphological characterization.
- Topological Dataset ($D_{T}$): Comprises 900 PD tests, of which 300 are original PD tests and 600 are synthetically augmented. These tests are organized into three groups according to label complexity, as shown in Table 3.

#### 4.1.2. Data Augmentation

To generate multi-label scenarios from the original single-source PD tests, a data augmentation procedure was applied. New PD tests were created by concatenating discharge events—from a single source type or from different source types—with stochastic interference, producing noisy single-source and multi-source configurations, respectively. The multi-hot labels were updated according to the source types present in each augmented test. These multi-source tests were produced by superposing discharge events drawn from distinct single-source acquisitions; while this construction preserves the morphological signature and phase-resolved structure of each constituent source, it does not reproduce the electromagnetic interaction that may arise between sources physically coexisting in the same apparatus, so validation on genuinely coexisting multi-source discharges is left to future work.

In addition to concatenation, two stochastic transformations were applied: random removal of up to 30% of the events, and small random phase shifts drawn uniformly from the interval $\left[-3^{\circ },+3^{\circ }\right]$. These operations simulate natural variations in sensor triggering and acquisition timing while preserving the morphological signatures and phase-resolved structure of the discharges.

The resulting augmented PD tests were incorporated exclusively into the topological dataset ($D_{T}$), enabling training and evaluation of the framework on cases involving simultaneous PD source types. The stratified 80/10/10 partition was defined at the level of physical sources prior to augmentation: all PD tests derived from a given source, whether original or augmented, were assigned to the same partition, and multi-source tests were formed only by combining sources from the same partition. As no physical source appears in more than one of the training, validation, and test sets, the partition is free of data leakage by construction.

#### 4.1.3. Cross-Domain Evaluation Dataset

The external dataset used for cross-domain evaluation [33] was acquired at the High-Voltage Laboratory of the Federal University of Campina Grande (Campina Grande, Brazil), and was not used during training or validation of any model; all methods processed it exclusively at inference time with frozen parameters.

The dataset comprises 347 acquisitions organized into five test configurations: corona discharge in two needle-to-plane arrangements (grounded needle with 97 acquisitions and grounded plane with 100 acquisitions), surface discharge on a suspension insulator under simulated rain (50 acquisitions), and internal discharge in two dielectric media—a solid phenolic-plate cell (50 acquisitions), and a liquid oil reservoir with needle electrodes (50 acquisitions). Each acquisition consists of a 35 ms time window of simultaneously measured applied voltage and discharge current, captured using a high-frequency current transformer (HFCT) with an approximately linear response from 1 MHz to 80 MHz and sampled at 125 MSa/s.

To convert the raw continuous acquisitions into event-level representations compatible with the proposed pipeline, the following preprocessing procedure was applied. Individual PD events were detected by applying an amplitude threshold of six times the standard deviation (6σ) of the background signal. For each detected event, a waveform segment of 5 μs was extracted around the triggering instant. The extracted segments were then resampled from the original 125 MSa/s to 100 MSa/s, yielding 500-sample waveforms $x_{i}\in R^{500}$ consistent with the input specification of the morphological network. The corresponding phase angle $\phi_{i}$ was determined from the simultaneously recorded voltage channel, and the peak amplitude $A_{i}$ was computed from the resampled waveform.

After preprocessing, the five test configurations were grouped into the three-class label space used throughout this work: the two corona arrangements were merged under the corona label and the two internal media under the internal label, while the surface configuration retained the surface label. Since each resulting PD test contains a single PD source type without intentional noise injection, this dataset provides a single-label evaluation scenario under cross-domain conditions. Table 4 summarizes the resulting cross-domain evaluation dataset ($D_{\mathrm{CD}}$).

### 4.2. Training Details

Optimization followed the modular training procedure established in Section 3.5; this subsection reports the hyperparameters of the two trainable stages (I and III).

All models were trained and evaluated on a single workstation equipped with an Intel Core i9-12900K processor (Intel Corporation, Santa Clara, CA, USA), 32 GB of RAM, and an NVIDIA GeForce RTX 3050 GPU (NVIDIA Corporation, Santa Clara, CA, USA) with 8 GB of memory. The proposed framework and all baselines were implemented in Python 3.10 with PyTorch 2.3 and PyTorch Geometric 2.5 under CUDA 12.1; the conventional machine learning baselines relied on scikit-learn 1.4, as did the clustering and evaluation routines. The same hardware and software environment was used for the inference cost and scalability measurements reported in Section 5.6.

#### 4.2.1. Stage I: Morphological Network Training

The multi-task neural network for morphological characterization is trained for up to 500 epochs using the AdamW optimizer with an initial learning rate of $1\times 10^{-4}$, weight decay of $1\times 10^{-2}$, and batch size of 256 waveforms. A cosine annealing with warm restarts scheduler is applied, configured with an initial period $T_{0}=10$ epochs, multiplication factor $T_{\mathrm{mult}}=2$, and minimum learning rate $\eta_{\min}=1\times 10^{-6}$.

The optimization objective combines a triplet loss and a focal loss (γ=2.5, α=0.5) for the embedding and confidence heads, respectively, with a balancing coefficient λ=0.1. The triplet loss employs a semi-hard mining strategy with a margin of m=0.3. An early-stopping criterion monitors the validation loss with a patience of 50 epochs. Gradient clipping is applied with a maximum norm of 1.0.

#### 4.2.2. Stage II: Graph Construction Parameters

Table 5 summarizes the hyperparameters governing the graph construction procedure described in Section 3.3. These values remain fixed across all in-domain experiments; the only exception is the minimum number of retained events, which is relaxed to one for the cross-domain evaluation (Section 5.4), where the per-test pulse counts are substantially lower. Their selection is supported by the sensitivity analysis presented in Section 5.5.

#### 4.2.3. Stage III: Graph Classifier Training

Upon freezing the morphological network weights, the edge-conditioned graph classifier is trained for up to 500 epochs. The optimization utilizes AdamW with a learning rate of $1\times 10^{-3}$ and weight decay of $5\times 10^{-3}$. Due to the variable density of the PD tests, a gradient accumulation strategy is adopted with a factor of 16 steps and a batch size of 1, resulting in an effective batch size of 16 graphs. The classification output is optimized using binary cross-entropy loss with label smoothing of 0.05 to support the multi-label formulation. A cosine annealing scheduler is used with $\eta_{\min}=1\times 10^{-6}$ along with an early-stopping patience of 100 epochs, gradient clipping with a maximum norm of 1.0, and a dropout rate of 0.5. Sinusoidal positional encodings of 64 dimensions are concatenated with the three-dimensional node features to provide spatial context within the phase-amplitude domain, yielding an input dimensionality of $d_{in}=67$ for the projection layer.

### 4.3. Baseline Methods for Benchmarking

To establish a comparative analysis, the proposed framework is evaluated against two groups of baselines. The first group comprises three histogram-based paradigms representative of the state of the art in PD source-type classification—grid-based deep learning, conventional machine learning with manual feature engineering, and feedforward neural networks without structural inductive biases—and operates on the phase-amplitude representation of each PD test. The second group comprises two event-level set-based controls of contrasting capacities: DeepSets [35], a pooling-only encoder, and the Set Transformer [36], an attention-based encoder. Both of these consume the same Stage I outputs (the per-pulse morphological embeddings and confidence scores) but aggregate the events as an unordered set rather than through the waveform-informed graph. By bracketing the spectrum from pooling-only aggregation to learned cross-event attention, these two controls isolate the contribution of the graph: they receive the morphological embeddings directly as event features and share the training recipe of the proposed framework, meaning that any performance difference is attributable to the relational structure rather than access to waveform-level information. All methods are trained and evaluated on the same topological dataset ($D_{T}$) under the same stratified partitions, and are subsequently applied to the cross-domain evaluation dataset ($D_{\mathrm{CD}}$) at inference time with frozen parameters, using the input representations and decision thresholds established during training. Existing graph-based methods for PD analysis [28,31,32] are not included as baselines, as they address different tasks (semi-supervised recognition, defect identification, binary PD detection) at different granularities (image patches, full measurement signals) and as such are not directly applicable to the multi-label PD source-type classification problem considered in this work.

#### 4.3.1. Grid-Based Convolutional Neural Network

This model represents the grid-based deep learning paradigm, where PD activity is treated as a dense image. The discharge events from the topological dataset are transformed into a phase-amplitude accumulation matrix with dimensions of 72×50 bins. To emulate the peak detector behavior of the original acquisition hardware, the absolute value of the raw amplitudes is used during discretization. The architecture consists of a five-layer convolutional neural network with 101,571 learnable parameters. The output layer is adapted with a sigmoid function to support the multi-label formulation.

#### 4.3.2. Conventional Machine Learning via SVMs

Two SVM variants are implemented over the flattened PRPD histogram, where the normalized event counts are recorded for each phase-amplitude bin. A linear kernel and a radial basis function (RBF) kernel are evaluated to establish both linear and nonlinear decision boundaries. A grid search procedure with three-fold cross-validation is employed to determine the penalty factor *C* and the kernel coefficient for each model.

#### 4.3.3. Deep Feedforward Neural Network

A feedforward neural network (FNN) without structural inductive biases is employed as a reference for classical neural models. The network is configured with four layers and 2,010,115 learnable parameters. The input data are presented in a flattened format, requiring the model to learn correlations between phase and amplitude without spatial filters or relational structures. The optimization is conducted using the AdamW optimizer with a learning rate determined through grid search.

#### 4.3.4. DeepSets

This control implements the canonical permutation-invariant set encoder of Zaheer et al. [35], adapted to the multi-label PD source-type classification setting. Each retained discharge event is represented by concatenation of its Stage I morphological embedding $h_{i}\in R^{32}$ with its normalized phase-amplitude coordinates and confidence $\left[{\tilde{\phi }}_{i},{\tilde{A}}_{i},p_{i}\right]$, yielding a 35-dimensional per-event feature vector. A shared event-wise multilayer perceptron with three hidden layers of 512 units projects each event independently, after which the concatenation of mean and max pooling produces a permutation-invariant set descriptor; a second multilayer perceptron with dropout (0.3) maps this descriptor to three independent logits. This training recipe consisting of optimizer, learning rate, weight decay, scheduler, effective batch size, gradient clipping, and binary cross-entropy with label smoothing of 0.05 replicates the one specified for Stage III of the proposed framework (Section 4.2), and node filtering reuses the same confidence threshold $\tau_{p}$; as a result, this baseline differs from the proposed framework in two coupled respects: it omits the graph construction and message passing, and it supplies each event’s morphological embedding $h_{i}$ directly as a node feature, whereas the proposed framework exposes $h_{i}$ to the network only through the morphological edge weight $W_{\mathrm{morph}}$. Therefore, the set control retains more direct nodal access to the waveform morphology than the graph framework, meaning that the comparison provides a conservative estimate of the contribution of the relational structure.

#### 4.3.5. Set Transformer

This control implements the Set Transformer of Lee et al. [36], an attention-based permutation-invariant set encoder. Each event is represented by the same 35-dimensional vector used in the DeepSets control and is first projected to a 96-dimensional latent space. Two stacked induced set attention blocks (ISAB), each with two attention heads and sixteen inducing points, model dependencies between events with linear rather than quadratic complexity; a single-seed pooling by multihead attention then produces a permutation-invariant set descriptor, which is mapped to three independent logits by a final multilayer perceptron with dropout (0.2). The capacity ($d_{\mathrm{model}}=96$, two ISAB blocks, sixteen inducing points) was selected on the validation set, where larger configurations were observed to overfit and exhibit greater seed-to-seed variance. The training recipe matches that of the DeepSets control except for a smaller learning rate of $3\times 10^{-4}$ and a 20-epoch grace period before the early-stopping criterion was applied, both of which were selected on the validation set to stabilize the attention blocks during the initial training phase. Unlike the DeepSets control, the events are aggregated through global self-attention rather than pooling; as in DeepSets, however, it departs from the proposed framework in the same two coupled respects, namely, the absence of the waveform-informed graph construction and the use of the morphological embedding $h_{i}$ as a direct node feature rather than through the edge weighting, meaning that it likewise provides a conservative estimate of the contribution of the relational structure.

## 5. Results and Discussion

This section reports the experimental evaluation of the proposed framework. It first characterizes the quality of the Stage I morphological representation, that is, the ability of the confidence head to separate valid PD pulses from stochastic interference and of the learned embeddings to organize the discharge sources in the latent space. It then evaluates multi-label classification performance within the training domain against the grid-based and set-based baselines to identify the conditions under which the explicit relational structure contributes most, and analyzes the residual misclassifications by type, acquisition scenario, and decision confidence. Generalization is assessed on a cross-domain dataset acquired with different instrumentation and test object geometries, with all parameters kept frozen. An ablation and sensitivity analysis subsequently isolates the contribution of the individual graph construction mechanisms and examines the robustness of the framework to its principal hyperparameters and architectural choices. The section closes with a characterization of the inference cost and scalability of the framework relative to the baselines.

The statistical analysis in this section follows two conventions. First, unless stated otherwise, every metric is reported as the mean over five independent training seeds, and the value following the ± sign is the half-width of the 95% confidence interval (CI95) of that mean across the seeds. Second, the significance of the difference in MCC between the proposed framework and a given baseline is assessed with a paired trial-level bootstrap evaluated on the per-test predictions of a single reference seed, fixed a priori as the first of the five initialization seeds so as to avoid any post hoc seed selection. For each pairwise comparison, the PD tests of the evaluation set are resampled with replacement over B=10,000 iterations; in every iteration, the MCC of both methods is recomputed on the same resampled set of tests so that the comparison is paired on the tests, and the difference $\Delta \mathrm{MCC}={\mathrm{MCC}}_{\mathrm{proposed}}-{\mathrm{MCC}}_{\mathrm{baseline}}$ is recorded to yield a bootstrap distribution of ΔMCC from which a two-sided *p*-value is derived. Because the proposed framework is compared against the six baselines simultaneously, these per-comparison *p*-values are adjusted for multiplicity with the Holm–Bonferroni procedure and reported as $p_{\mathrm{Holm}}$. To confirm that significant gaps are not artifacts of the reference seed, the same trial-level bootstrap is additionally applied within each of the five seeds, and we report the number of seeds attaining significance at α=0.05.

### 5.1. Evaluation of Morphological Embeddings and Confidence Scores

The graph construction and classification stages rely on the outputs of the multi-task morphological network, which generates confidence scores for node filtering and edge gating; the raw high-frequency PD waveforms are mapped to a 32-dimensional latent space for relational connectivity. Table 6 summarizes the performance of the confidence and embedding heads.

#### 5.1.1. Confidence Scores and Gating

**Figure 4:**
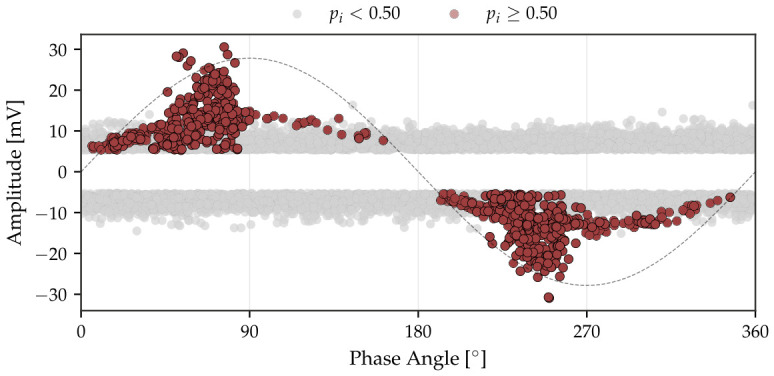
Phase-amplitude projection of a representative PD test with events classified by the confidence head at the threshold $\tau_{p}=0.5$. The dashed sinusoid indicates the AC reference waveform. Valid PD pulses ($p_{i}\geq 0.5$, red markers) are separated from stochastic interference ($p_{i}<0.5$, grey markers) based on waveform morphology, even in regions where both overlap in amplitude and phase.

The confidence head trained with focal loss produces per-pulse scores $p_{i}$ that are incorporated into the graph through the gating weight. At the fixed threshold $\tau_{p}=0.5$ used throughout the framework, this head achieves an area under the receiver operating characteristic curve (AUROC) of 0.9996±0.0003 and a Matthews correlation coefficient (MCC) of 0.9884±0.0046, with no recalibration of the threshold required across seeds or PD source types. Figure 4 illustrates the separation capability of the confidence head on a representative PD test projected onto the phase-amplitude plane. The head distinguishes valid PD pulses from stochastic interference based on waveform morphology, even when interference overlaps with valid PD activity in both amplitude and phase. The resulting gating weights attenuate the contribution of low-confidence events during message passing, ensuring that global aggregation relies primarily on valid discharge events.

#### 5.1.2. Manifold Structure

**Figure 5:**
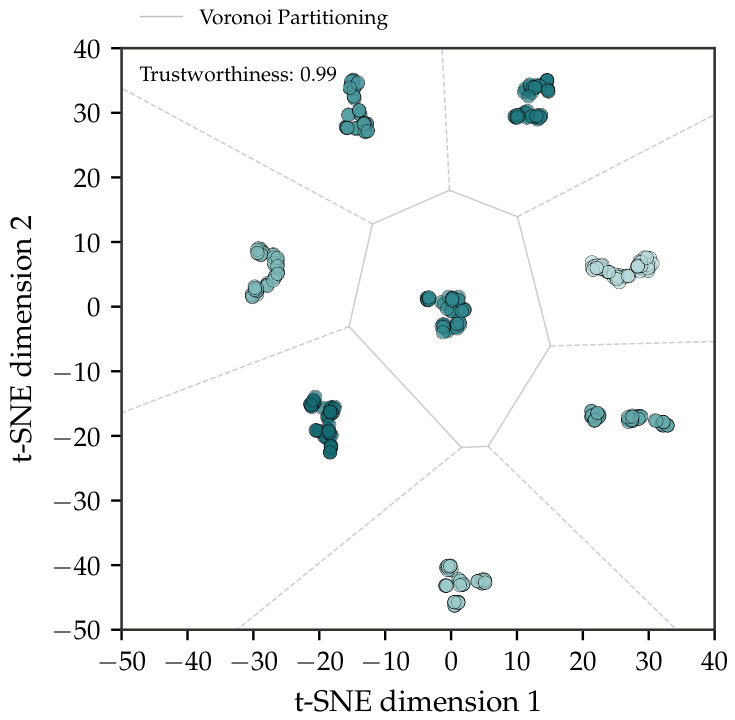
t-SNE projection of the morphological embeddings on the test subset, with dashed grey lines indicating the overlaid Voronoi partitioning of the source centroids. Samples from the same physical source cluster together, confirming that the learned latent space preserves source-level separability. The trustworthiness score of 0.99 indicates high preservation of local neighborhood structures in the 2D projection.

The 32-dimensional latent space $h_{i}$ yields a cosine Silhouette coefficient of 0.9121±0.0178, indicating compact and well-separated clusters at the level of physical sources. The normalized mutual information (NMI) of 1.0000±0.0000 confirms that the learned manifold aligns with the physical sources. This organization is illustrated in Figure 5, which presents the t-distributed stochastic neighbor embedding (t-SNE) projection of the test samples. A trustworthiness score of 0.99 further validates that local neighborhood structures are preserved in the projection, providing a reliable basis for the morphological similarity filter used during graph construction.

**Figure 6:**
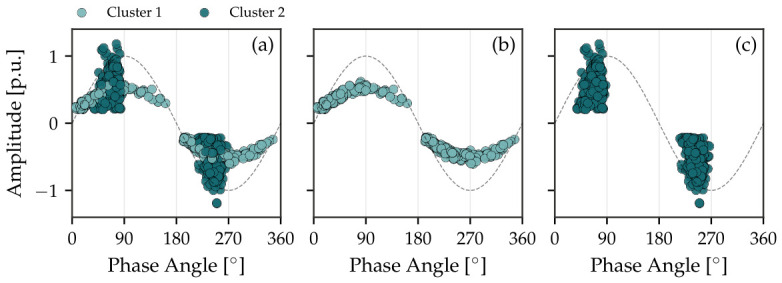
Phase-amplitude projection of a representative multi-source PD test from the test subset of $D_{T}$, with each pulse colored by the source assignment recovered through *k*-means clustering of the morphological embeddings. (**a**) Both sources overlaid; (**b**,**c**) each source shown separately (light and dark markers, respectively). The dashed sinusoid indicates the AC reference waveform. The two sources overlap in the phase-amplitude plane yet are cleanly separated by the embedding-based assignment, illustrating the source-level separability of the learned latent space.

**Figure 7:**
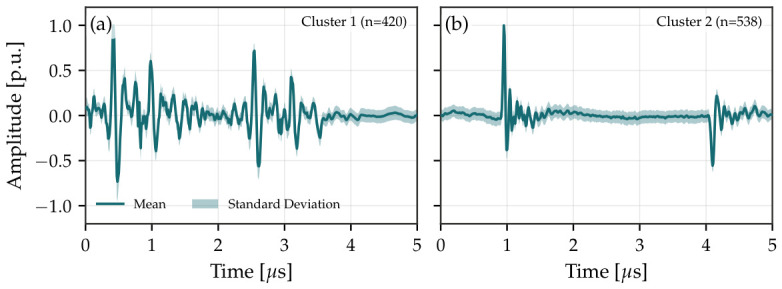
Mean waveform (solid line) and standard deviation (shaded band) of the high-frequency PD waveforms assigned to each cluster of the representative multi-source PD test shown in Figure 6; the waveforms were normalized and aligned by their peak amplitude. (**a**) Cluster 1 (n=420 pulses) and (**b**) Cluster 2 (n=538 pulses). The two clusters present distinct transient signatures, providing the waveform-level basis for the source separation observed in the phase-amplitude plane.

The practical consequence of this latent-space organization is the ability to disentangle PD sources that coexist within a single test. Figure 6 shows a representative multi-source test from $D_{T}$ in the phase-amplitude plane, with each pulse colored by the source assignment recovered through *k*-means clustering of its morphological embedding. The two sources occupy overlapping regions of the phase-amplitude plane—a configuration in which phase-binning grid representations cannot isolate the individual contributions—yet the embedding-based assignment recovers coherent per-pulse source membership, distinguishing the two sources from their waveform morphology alone. The morphological basis for this separation is made explicit in Figure 7, which overlays the mean waveform and standard deviation of the events assigned to each cluster: the two clusters exhibit distinct transient signatures, the first a prolonged oscillatory transient and the second a sharper more compact pulse, confirming that the separation seen in the phase-amplitude plane reflects a genuine difference in high-frequency waveform shape rather than an artifact of the embedding-based assignment. This disentanglement capability, rooted in the latent-space organization discussed above, is the property that the morphological similarity filter exploits during graph construction; its contribution to multi-label classification is quantified by the component ablation in Section 5.5.

Taken together, these results admit a direct physical reading of the constructed graph at each level. The nodes correspond to physically valid discharges. The confidence head retains genuine PD pulses and rejects stochastic interference on the basis of waveform morphology (Figure 4), so that the graph is built over events that carry diagnostic information rather than over noise. The edges encode morphological consistency between events; because the latent space organizes pulses by physical source (Figure 5, with the source-level separation of Figure 6), the morphological similarity weight $W_{\mathrm{morph}}$ is high between discharges that share a high-frequency signature and low between discharges that do not, even when the latter coincide in phase and amplitude. Message passing inherits this structure; because $W_{\mathrm{morph}}$ enters the edge attribute that conditions the Edge-MLP (Section 3.4), the network modulates the exchange of information according to waveform-signature consistency, aggregating preferentially over morphologically coherent (and consequently source-coherent) neighborhoods. The functional necessity of this reading, rather than its mere plausibility, is established quantitatively by the component ablation discussed in Section 5.5, in which replacing the morphology-informed connectivity with a plain geometric *k*-NN graph collapses performance to the level of the grid-based baseline.

### 5.2. Classification Performance

The classification performance of the proposed graph-based framework was evaluated across three test conditions of increasing complexity from the topological dataset $D_{T}$: single-source without noise, single-source with noise, and multi-source configurations. The framework was compared against the histogram-based baselines (Grid-CNN, Deep FNN, Linear SVM, and Nonlinear SVM) and the event-level set-based controls (DeepSets and Set Transformer) introduced in Section 4.3. Table 7 summarizes the aggregated performance across all test conditions, quantified using AUROC, MCC, and the exact match ratio (EMR). Table 8 resolves the MCC across the three individual test conditions. The significance of the gap in MCC between the proposed framework and each baseline is assessed by the paired trial-level bootstrap described in the introduction of this section; for all baselines, the observed gap is positive with $p_{\mathrm{Holm}}<0.001$ after correction for multiple comparisons.

As reported in Table 8, the proposed framework maintained stable performance across all three scenarios, with its MCC decreasing only marginally from 0.99 in the clean single-source condition to 0.97 in the multi-source configuration. In the clean single-source scenario, several neural baselines reached comparable or saturated MCC; the framework’s advantage emerges as the test conditions become more demanding. Grid-CNN, which was competitive in the clean condition (MCC of 0.93±0.07), dropped to 0.64±0.05 under stochastic interference and 0.61±0.19 in the multi-source scenario. This degradation is consistent with the binning mechanism of the accumulation matrix: when events from distinct source types overlap in the phase-amplitude domain, their contributions are merged into shared bins, erasing the distributional signatures that the convolutional filters rely upon. The Deep FNN and SVM variants, which operate on flattened versions of the same histogram, exhibited MCC values between 0.44 and 0.60 in the multi-source condition, as the lack of any relational structure prevents them from disentangling superimposed sources.

The two event-level set-based controls share the proposed framework’s Stage I morphological embeddings and confidence scores, but consume them as an unordered set rather than as a relational graph; this design isolates the contribution of the explicit graph structure from that of the underlying waveform-level features. DeepSets remained comparable to the histogram baselines in the clean single-source condition (0.92±0.04) and degraded only marginally under stochastic interference (to 0.84±0.09), in contrast to the Grid-CNN, for which the accumulation-matrix representation dropped to 0.64±0.05. Among the baseline methods, the two set-based controls exhibited the smallest degradation from the clean to the noisy condition, consistent with the resilience that the per-pulse morphological features confer against stochastic interference. Set Transformer followed the same pattern in the single-source conditions but collapsed to 0.28±0.09 in the multi-source scenario, well below all other baselines, likely because its induced set-attention layers tend to homogenize the representation of co-occurring sources in the absence of explicit relational structure rather than disentangling them. The gap between the proposed framework and the stronger set-based control (DeepSets) is concentrated in the multi-source condition (ΔMCC=0.35) and statistically significant at α=0.05 in 5/5 seeds (against 0/5 in the single-source scenario), indicating that the contribution of the relational structure manifests specifically where overlapping discharge activity is most prevalent. Overall, these results attribute the framework’s advantage to two complementary mechanisms: the per-pulse morphological features, which are shared with the set-based controls, provide resilience to stochastic interference, while the explicit waveform-informed graph construction provides the disentanglement capability observed in the multi-source condition. In combination, they sustain an MCC of at least 0.97 across all three test conditions.

### 5.3. Error Analysis

**Figure 8:**
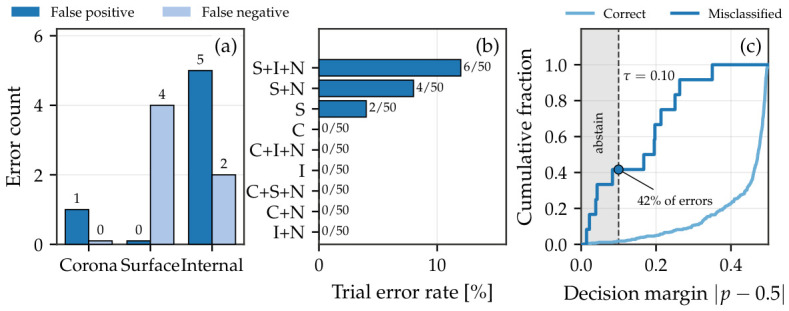
Aggregate error analysis over all 450 trial-level decisions on the test subset of $D_{T}$ (5 seeds × 90 tests). (**a**) Per-class error type, decomposed into false positives and false negatives. (**b**) Trial-level error rate by acquisition scenario, where a trial is counted as erroneous if any class is misclassified; scenario codes denote corona (C), surface (S), internal (I), and injected noise (N). (**c**) Cumulative distribution of the decision margin |p−0.5| for correctly classified and misclassified trials, with the shaded region indicating the abstention zone of a selective prediction rule at τ=0.10.

The aggregate metrics of Section 5.2 are complemented here by an analysis of the residual misclassifications on the in-domain test set, characterizing their type, their distribution across acquisition scenarios, and the confidence of the model when it errs. The analysis pools every per-test decision across the five seeds, yielding 5×90=450 trial-level decisions; each PD test is reconstructed into its relational graph from the raw pulses along the same evaluation path used in Section 5.2, meaning that the errors examined here correspond exactly to the reported event-level metrics. Figure 8 summarizes this view.

Of the 450 decisions, 12 were misclassified, a trial-level error rate of 2.7%, and every one of them occurred in a scenario containing surface discharge: the surface–internal multi-source condition (S + I + N, six errors out of 50 trials), the noisy single-source surface condition (S + N, four out of 50), and the clean single-source surface condition (S, two out of 50). The remaining six scenarios were classified without error (Figure 8b), including the corona–surface multi-source condition (C + S + N). Each misclassified trial differed from its ground truth in exactly one class. The per-class decomposition (Figure 8a) localizes the residual errors to the surface and internal classes: corona was never missed (zero false negatives) and produced a single false positive, surface accounted for four false negatives and no false positives, and internal accounted for five false positives and two false negatives.

The scenario localization further constrains the interpretation of these errors. Because internal discharge is absent from the single-source surface conditions by construction, the five internal false positives necessarily arose there: five of the six errors in the single-source surface conditions (S and S + N) were false-positive internal detections. In contrast, the two internal false negatives could only have occurred in the surface–internal multi-source condition, where internal discharge is genuinely present. Together with the absence of any surface false positive and any corona false negative, this indicates a bidirectional confusion confined to the surface–internal pair: internal is over-predicted in a subset of single-source surface trials, and one of the two co-occurring sources is occasionally missed in the surface–internal mixtures. Corona, for which the single-polarity low-amplitude morphology is the most distinct of the three types, was never missed and was recovered without error even when superimposed on surface discharge. This pattern is consistent with the cross-domain per-class results presented in Section 5.4, in which the internal class likewise exhibited the lowest MCC.

The decision margin |p−0.5|, defined per trial as the smallest absolute distance of any class probability from the 0.5 threshold, separates the misclassified from the correctly classified trials (Figure 8c): the cumulative distribution of the misclassified trials is concentrated at substantially smaller margins, indicating that the misclassifications fall predominantly among the lowest-confidence decisions. A selective prediction rule that abstains on the lowest-margin decisions exploits this separation; abstaining at a margin threshold of τ=0.10 flags 42% of the errors at the cost of deferring only a small fraction of the correct trials. This behavior is operationally relevant for field diagnostics, where low-margin trials can be routed for expert review rather than being acted upon automatically.

### 5.4. Cross-Domain Generalization

To evaluate the generalization capability of the proposed framework beyond the training domain, all methods were applied to the cross-domain evaluation dataset ($D_{\mathrm{CD}}$) described in Section 4.1.3. No model was retrained or fine-tuned; all parameters remained frozen at the values obtained from in-domain training. For the graph-based framework, the minimum-nodes filter applied during graph construction was relaxed from its in-domain default to a single node, ensuring that every acquisition in $D_{\mathrm{CD}}$—including those with very few valid pulses after Stage I gating—was represented as a valid graph and included in the evaluation. Table 9 reports the overall AUROC, MCC, and EMR of all methods, and Table 10 resolves the MCC by PD source type.

The proposed framework achieved the strongest overall performance under the domain shift, with an MCC of 0.93±0.04, AUROC of 1.00±0.00, and EMR of 0.94±0.03; its per-class MCC stayed between 0.87 (Internal) and 0.99 (Surface), demonstrating consistent generalization across a domain shift involving different laboratory equipment and test-object geometries. The baseline methods diverged from this behavior in two distinct ways. The histogram-based baselines suffered a generalized degradation driven by changes in pulse density: Grid-CNN collapsed to an MCC of −0.29±0.05 with an AUROC of 0.38±0.06, below the 0.5 chance level, and Nonlinear SVM reached a near-zero MCC of −0.02±0.01. Meanwhile, Deep FNN and Linear SVM fell to 0.61±0.07 and 0.60±0.01, well below their in-domain results shown in Table 7. The event-level set-based controls proved markedly more robust in the aggregate, retaining MCC values of 0.89±0.09 (Set Transformer) and 0.80±0.03 (DeepSets), although this aggregate resilience conceals a strong class dependence examined below.

The significance of the per-method MCC gap relative to the proposed framework was assessed with the paired trial-level bootstrap described in the introduction of this section. The gap was positive and significant after Holm–Bonferroni correction against every histogram baseline ($p_{\mathrm{Holm}}<0.001$ for Grid-CNN, Deep FNN, Linear SVM, and Nonlinear SVM) and against DeepSets ($p_{\mathrm{Holm}}<0.001$). Against Set Transformer, the difference was not significant ($p_{\mathrm{Holm}}=0.64$); thus, the two methods are statistically comparable in aggregate MCC under the domain shift. Because this aggregate gap was not significant, the per-seed robustness check targeted DeepSets, the set-based control against which the framework showed a significant aggregate gap; the MCC gap there remained significant at α=0.05 in four of the five seeds, the single exception being a seed in which it narrowed to +0.05 (p=0.11).

#### 5.4.1. Grid-CNN Failure Analysis

The complete failure of Grid-CNN, with near-zero or negative per-class MCC on all three classes (Table 10) and a negative overall MCC, is explained by the distributional mismatch between the training and cross-domain data in the grid representation. For each PD source type, Table 11 compares the average number of detected pulses and corresponding bin occupancy of the 72×50 accumulation matrix used by Grid-CNN. The $D_{T}$ values are computed as the per-class average across the single-source and noisy single-source conditions, providing a representative reference for each discharge type during training.

The comparison reveals a substantial distributional mismatch. Corona acquisitions from $D_{\mathrm{CD}}$ occupy only 0.8% of the grid bins compared to 19.1% for the corresponding training data, a reduction factor of approximately 24×. Internal acquisitions show the same pattern (2.8% versus 18.8%). Under these conditions, the convolutional feature extractor operates far outside its training regime: the batch-normalization statistics calibrated on denser maps become misaligned with the sparse inference distributions and the global average pooling over largely empty feature maps yields uninformative descriptors, driving predictions to a degenerate regime. Even the Surface class, which exhibits the highest pulse density in $D_{\mathrm{CD}}$ at 11.0% occupancy, remains below the 14.7% observed during training; therefore, no class in $D_{\mathrm{CD}}$ reaches the bin-occupancy regime on which Grid-CNN was calibrated, consistent with its negative MCC and below-chance AUROC.

#### 5.4.2. Resilience of the Graph-Based Framework

The proposed framework maintains robust performance under domain shift, in part because its representation does not depend on the density of an accumulation grid. The morphological network processes each waveform individually, extracting per-pulse embeddings and confidence scores that characterize the high-frequency signature of the event independently of the total number of pulses in the PD test. The graph construction stage then establishes connectivity based on spatial proximity and morphological similarity among the retained events, producing a topological structure that adapts naturally to the number of available nodes. Consequently, a PD test with 30 pulses generates a smaller but structurally valid graph, whereas the same 30 pulses projected onto a 72×50 grid produce a representation that falls outside the training distribution of the CNN.

Deep FNN and Linear SVM, which also operate on the flattened grid representation, exhibited intermediate degradation rather than collapse: their dense and linear decision functions degraded gradually as the histograms become sparser, leaving them well below their in-domain accuracy but above a negative MCC. In contrast, Nonlinear SVM collapsed to a near-zero overall MCC because its radial basis function kernel is local—the cross-domain samples, lying far from the training support vectors in feature space, elicit near-uniform kernel responses and degenerate decisions. Across all four grid-based methods, the overall MCC stayed at or below 0.61 and the per-class MCC on the Internal class stayed below 0.55 for every one of them, confirming that the histogram representation limits cross-domain generalization regardless of the specific classifier head.

The event-level set-based controls operate independently of any accumulation grid and consume the same per-pulse morphological embeddings as the proposed framework, accordingly avoiding the distributional mismatch that affects the histogram-based baselines; their aggregate MCC values place them well above the histogram methods. However, the per-class breakdown in Table 10 exposes a contrast that the aggregate values obscure. The strong aggregate MCC of the set-based controls rested heavily on the most populous class, Corona (n=197), on which both were near-perfect (1.00). They degraded sharply on the smallest class, Surface (n=50), with Set Transformer falling to 0.65±0.32 and DeepSets to 0.16±0.28; DeepSets also dropped on the Internal class, to 0.74. The confidence intervals on the Surface class was more than an order of magnitude wider than the framework’s ±0.02, showing that the set-based controls are not only less accurate there but are markedly less stable; they account for the larger aggregate variance of Set Transformer (±0.09) relative to the proposed framework (±0.04). By maintaining MCC between 0.87 and 0.99 on every class, the proposed framework is the only method that combines grid-independence with per-class consistency across the domain shift; its explicit waveform-informed graph structure stabilizes performance across classes in a way that permutation-invariant aggregators do not. In aggregate MCC, the proposed framework and Set Transformer are statistically comparable, as established above; however, the per-class evidence—most obviously on the Surface class—identifies our framework as the more reliable of the two under the domain shift.

Two scope considerations apply to these results. First, the domain shift evaluated here combines variation across acquisition hardware (sensor type, signal conditioning, sampling configuration), laboratory environment, and test-object geometry; therefore, the cross-domain results constitute combined evidence of robustness to hardware variation in practice, but do not isolate the sensitivity of the framework to individual hardware parameters such as acquisition bandwidth, synchronization accuracy with the AC reference, or channel frequency response. Second, the cross-domain dataset $D_{\mathrm{CD}}$ contains only single-source PD tests; the robustness of the framework to multi-source configurations under domain shift was not evaluated, and the collection of a multi-source cross-domain dataset is identified as a direction for future work.

### 5.5. Ablation and Sensitivity Analysis

This subsection presents three complementary analyses. The first evaluates the sensitivity of the framework to the key hyperparameters governing the morphological characterization and graph construction stages, the second quantifies the contribution of the morphological embeddings and confidence scores to the overall classification performance, and the third examines the influence of the backbone depth and pooling strategy on the quality of the encoder’s outputs. The first two analyses are conducted on the topological dataset $D_{T}$ using the same training, validation, and test partitions as the main experiments, and report the MCC. The third is conducted on the morphological dataset $D_{M}$ and reports three of the Stage I metrics introduced in Section 5.1. All results are reported as mean ± CI95 over five seeds.

#### 5.5.1. Hyperparameter Sensitivity

Five hyperparameters were evaluated: embedding dimensionality *d*, number of nearest neighbors *k*, confidence threshold $\tau_{p}$, morphological similarity threshold $\tau_{\mathrm{morph}}$, and morphological exponent γ. In each experiment, a single hyperparameter was varied while all others were held at their default values (Table 5; the embedding dimensionality was at its default of d=32). Results are summarized in Table 12.

The embedding dimensionality *d* affects the capacity of the latent space to represent morphological variability. At d=16, the reduced capacity limits the separation between physical sources, resulting in a decrease of 0.04 relative to d=32. Increasing to d=64 introduces redundancy without proportional gain and yields a decrease of 0.02, consistent with overfitting to the available training data in $D_{M}$.

For the number of nearest neighbors *k*, performance peaks at k=5 and decreases for both lower and higher values. At k=3 the graph becomes overly sparse, limiting the receptive field of the message-passing layers. At k=10 the increased connectivity admits edges between morphologically dissimilar events, diluting the selectivity of the graph structure.

The confidence threshold $\tau_{p}$ controls the tradeoff between noise suppression and event retention. A permissive threshold ($\tau_{p}=0.3$) retains a larger proportion of stochastic interference, degrading classification by 0.05. A restrictive threshold ($\tau_{p}=0.7$) discards valid low-amplitude PD events, reducing performance by 0.02.

The morphological similarity threshold $\tau_{\mathrm{morph}}$ exhibits analogous behavior. At $\tau_{\mathrm{morph}}=0.1$, the filter admits edges between pulses with weak morphological correspondence, effectively reducing the construction to a geometric *k*-NN graph. At $\tau_{\mathrm{morph}}=0.7$, the overly restrictive criterion produces disconnected subgraphs for certain source types, resulting in a decrease of 0.06.

The morphological exponent γ modulates the contrast of the similarity weighting. At γ=1.0 the weighting is approximately linear, providing limited discrimination between moderate and high similarity values. At γ=2.0 the nonlinearity sharpens the contrast, and a further increase to γ=3.0 yields no additional benefit.

#### 5.5.2. Component Ablation

To quantify the contribution of the morphological and confidence information to the graph construction, four configurations were evaluated by progressively disabling the outputs of the multi-task morphological network while keeping all other parameters at their default values. Results are summarized in Table 13.

In the full framework, the multi-task morphological network supplies both the 32-dimensional embeddings $h_{i}$ (used for the hard morphological similarity filter with threshold $\tau_{\mathrm{morph}}$ and the soft edge weight $W_{\mathrm{morph}}$) and the per-event confidence scores $p_{i}$ (used for node filtering and the gating weight $W_{\mathrm{gate}}$).

Disabling morphological similarity by forcing $W_{\mathrm{morph}}=1.0$ for all candidate edges removes the hard similarity filter and sets all morphological edge weights to a constant value. This results in a decrease of 0.09, indicating that waveform-level morphology contributes to establishing structurally meaningful connectivity and to separating overlapping PD source types.

Disabling confidence filtering by setting $p_{i}=1.0$ for every event eliminates both node filtering and edge gating, causing a larger decrease of 0.14. Under this configuration all detected pulses, including stochastic interference, contribute equally during message passing.

When both mechanisms are removed simultaneously, the model operates on a conventional geometric *k*-NN graph built from all detected events. This yields the lowest MCC at 0.73±0.05, a reduction of 0.25 relative to the full framework. This result is consistent with the performance of the Grid-CNN baseline (MCC of 0.72±0.08), which further supports the interpretation that the classification advantage of the proposed framework stems primarily from the quality of the graph construction rather than from the GNN architecture alone. This configuration also provides the complement of the event-level set-based controls within a factorial comparison: here, the graph is retained while waveform-level information is removed, whereas the set-based controls (Section 4.3) remove the graph while retaining waveform-level information. The substantial reduction in MCC observed here together with the gap between the set-based controls and the full framework reported in Section 5.2 indicates that the framework’s performance requires the joint use of the explicit graph and waveform-level information rather than either alone.

#### 5.5.3. Backbone Architectural Variants

To support the architectural choices specified in Section 3.2, the backbone depth and pooling strategy of the morphological characterization stage were varied one factor at a time around the default configuration of three residual blocks and a dual-pooling head combining global average pooling and global max pooling. Each variant was trained on $D_{M}$ using the same recipe as the proposed framework, and the resulting Stage I metrics on the test subset (Silhouette coefficient (*S*), NMI, and gating MCC) were averaged over five seeds. The Stage III classifier was held fixed at its default configuration throughout, meaning that the variations reported here isolate the effect of the morphological backbone on the encoder’s representational quality and on the reliability of the gating head. The results are reported in Table 14.

The default configuration yielded the highest values across all three metrics, confirming that the architectural choices specified in Section 3.2 operate near the empirical optimum within the evaluated range. Reducing the backbone to two residual blocks degraded the Silhouette coefficient by approximately 0.06 and the NMI by 0.05 relative to the default, indicating that the resulting encoder produces less discriminative clusters in the latent space; the gating MCC showed a smaller but consistent decline. Increasing the backbone to four residual blocks did not yield further improvement and slightly increased the seed-to-seed variance, which is in agreement with the overfitting argument made in Section 3.2, i.e., the morphological dataset $D_{M}$ does not provide sufficient samples to support the additional capacity. The pooling variants exhibited a complementary pattern in which removing either global average pooling or global max pooling from the dual-pooling layer caused a comparable drop in Silhouette (of approximately 0.05 and 0.04, respectively) and a smaller but consistent reduction in the remaining metrics, corroborating the role of these two operations as carriers of complementary descriptors of the input waveform (average pooling for the envelope of the transient signal and the max pooling for the peak amplitude of the discharge event). The dual-pooling configuration retains both, thereby yielding the highest separability in the latent space.

Collectively, the three analyses indicate that the chosen configuration operates near its empirical optimum within the evaluated ranges. The scalar hyperparameters sit at the peaks of their respective sensitivity curves; the morphological embeddings and confidence scores provide complementary contributions, and their joint removal accounts for the largest performance degradation; finally, the most discriminative Stage I outputs are yielded by the backbone depth and the pooling strategy. The interaction of these components, rather than any one in isolation, is central to the robustness of the framework under stochastic interference and multi-source conditions.

### 5.6. Computational Characterization

This subsection reports the computational characterization of the framework along three dimensions: the topology of the constructed graphs across the scenarios of the topological dataset $D_{T}$, the inference cost of the proposed framework relative to the baselines on a representative test trial, and the scalability of the framework as the input size grows. All timings were measured on the same hardware and software stack as the training runs of Section 4.2 and are reported as the median over multiple repetitions on the same input to mitigate measurement noise.

#### 5.6.1. Graph Topology by Scenario

Table 15 reports the topology of the graphs constructed on the test subset of $D_{T}$, grouped by scenario.

The number of nodes *V* reflects the pulses that pass the gating threshold and morphological filter, while the number of edges *E* reflects the connections retained after the *k*-NN and morphological similarity steps. Multi-source scenarios produce the largest graphs on average (V≈715, E≈3567), as expected from the superposition of two underlying source types; single-source noisy scenarios produce the smallest (V≈430, E≈2142) despite having the highest raw pulse counts, which is because the gating mechanism removes the injected stochastic interference and leaves only the valid discharge pulses as nodes. The edge density remains within the range 0.7–1.3% across all scenarios, consistent with the bounded neighborhood structure (k=5) of the construction. Therefore, the graphs are both sparse and bounded in average degree, which is the precondition for the cost behavior reported in the next two sub-subsections.

#### 5.6.2. Per-Method Inference Cost

Table 16 reports the model size, total floating-point operations (FLOPs), and end-to-end inference latency of the proposed framework and the four neural baselines on a representative multi-source test trial of $D_{T}$ containing N=4622 detected pulses, where the retained graph has 879 nodes and 4359 edges.

The proposed framework incurs the highest absolute latency among the evaluated methods (≈117 ms), but operates well within the time scales required for online PD monitoring at the trial level (an effective throughput of approximately 8.5 Hz on the evaluated hardware). The dominant contribution is the per-pulse evaluation of the 1D-ResNet encoder in Stage I (64 ms, 55% of the total), followed by the graph construction in Stage II (43 ms, 37%); the Stage III GNN itself accounts for less than 10% of the cost (10 ms). The event-level set-based controls (DeepSets and Set Transformer) share the Stage I encoder with the proposed framework, leading to comparable contributions from the morphological forward pass, but replace the graph construction and message passing with a single permutation-invariant set aggregator, yielding lower total latency (65 and 68 ms). The difference is essentially the cost of the graph construction and message passing that they omit. The phase-resolved baselines (Grid-CNN and Deep FNN) operate on the precomputed PRPD matrix and have sub-millisecond costs for the forward pass, but require the PRPD rendering step (≈37 ms), which dominates their end-to-end latency. Therefore, the performance differential reported in Section 5.2 (an overall MCC of 0.98 for the proposed framework against 0.72 for Grid-CNN and 0.79 and 0.64 for the set-based controls) is obtained at an inference cost that remains compatible with online deployment. The additional cost is concentrated in the encoder and the graph construction rather than in the GNN itself.

#### 5.6.3. Scalability

To characterize how the framework behaves as the input size grows, the per-stage timings and the end-to-end latency were measured for input sizes ranging from N=100 to N=10,000 raw pulses, sampling each input from a real pulse pool aggregated from the highest-pulse-count acquisitions of $D_{T}$. These acquisitions are interference-laden, so the pool has a low fraction of valid pulses: after Stage I gating, only about 3.7% of the sampled pulses are retained as nodes. Therefore, alongside the raw input size *N*, Table 17 reports the median retained node count $N_{f}$ and edge count *E* which determine the cost of Stages II and III.

Three distinct regimes are observed. Stage I is asymptotically linear in the raw pulse count *N*; when batch-level parallelism becomes saturated (N≥1000), its cost is constant at ≈0.014 ms per pulse, with the higher apparent per-pulse cost at small *N* reflecting a fixed per-batch overhead rather than superlinear growth. Stage II scales sublinearly as approximately $N^{0.80}$ over the evaluated range, which is owing to the spatial indexing in the *k*-nearest neighbor step. Stage III is nearly flat (empirical exponent ≈0.09), dominated by a fixed cost of ≈3 ms and growing only at the largest graphs (5.1 ms at E=1855); this is because the message-passing cost depends on the number of edges per node, which is bounded by k=5 regardless of the total node count. The retained node and edge counts confirm the role of the gating; even a 10,000-pulse input yields only 371 nodes and 1855 edges, so the swept graphs are far sparser than a dense multi-source trial.

This last observation reconciles the present sub-subsection with the per-method cost of Table 16. Because the cost of Stages II and III is governed by the retained graph size ($N_{f}$, *E*) rather than by the raw pulse count, the higher Stage II and Stage III latencies of the representative trial—43.4 and 9.6 ms on its graph with 879 nodes and 4359 edges—are consistent with the per-stage trends of Table 17 extrapolated to that larger graph, and not with the equal-*N* rows, for which the graphs are an order of magnitude sparser. When keyed on retained nodes and edges, the two characterizations agree. The end-to-end latency is dominated by Stage I for large *N* and remains within ≈158 ms even for N=10,000 pulses, roughly twice the dataset-wide mean of ≈4633 detected pulses per trial (Table 3) and above the average pulse count of every individual scenario. Therefore, the framework scales gracefully and retains online-compatible latency well beyond the mean trial size, with the bounded growth of Stage III being a direct consequence of the bounded-degree topology established at construction.

## 6. Conclusions

This work presents a graph-based framework for multi-label classification of PD source types that combines the morphological characterization of raw high-frequency waveforms with the phase-amplitude coordinates of individual discharge events, retained at the event level rather than accumulated into a discretized PRPD pattern. The framework operates through three modular stages: a multi-task neural network that extracts per-pulse embeddings and confidence scores, a graph construction procedure that establishes selective connectivity based on spatial proximity and morphological similarity, and an edge-conditioned graph neural network that performs classification through message passing over the constructed graphs.

Experimental evaluation on PD measurements acquired in accordance with IEC 60270 demonstrated that the proposed framework achieved an MCC of 0.98 and an EMR of 0.97 across all test conditions, while the histogram-based baselines and the event-level set-based controls remain substantially behind. The advantage of the graph-based approach was most evident under conditions of increasing complexity: the proposed framework maintained an MCC of 0.97 in multi-source scenarios, where the gap relative to these methods was widest. Cross-domain evaluation on an external dataset acquired with different acquisition hardware, laboratory environment, and test-object geometry further demonstrated the robustness of the framework, which achieved an MCC of 0.93 without retraining, while Grid-CNN collapsed to a negative MCC due to the distributional mismatch in its grid representation. The ablation study confirmed the complementary contribution of the morphological embeddings and confidence scores: removing both components reduced the MCC by 0.25 to a level comparable with the grid-based baseline, indicating that our framework’s advantage stems primarily from the quality of its waveform-informed graph construction rather than from the graph neural network alone. These results suggest that graph-based representations operating at the level of individual discharge events can offer a viable alternative to grid-based approaches for PD diagnostics in condition-based maintenance programs, particularly in scenarios involving simultaneous PD source types and varying acquisition conditions.

Two limitations of the present evaluation outline direct avenues for future research. First, the cross-domain dataset $D_{\mathrm{CD}}$ comprised only single-source PD tests. Validating the framework’s signature disentanglement capability under domain shift—the regime in which its relational structure contributes most—will require the collection of a multi-source external dataset. Second, while the cross-domain results provide combined evidence of robustness to hardware variation, a dedicated controlled study is needed to isolate the framework’s sensitivity to individual acquisition parameters such as bandwidth, synchronization accuracy, and sensor frequency response.

Beyond addressing these experimental boundaries, several methodological extensions follow naturally from this work. Given that the per-test graph construction is well suited to online deployment, a logical next step is to replace the static construction with incremental streaming graph updates that revise the topology as new events arrive. Additionally, incorporating the temporal ordering and inter-cycle evolution of discharge activity through dynamic graph modeling could capture degradation trends that a single-snapshot representation does not. Finally, extending the framework to unseen PD source types through few-shot or meta-learning adaptation would broaden its diagnostic scope without full retraining, paving the way for its translation into industrial monitoring systems with strict on-device latency and memory constraints.

## Figures and Tables

**Table 1:** Architectural specifications of the multi-task morphological network.

Component	Specifications/Hyperparameters
Input Stem	Conv1D (k=7,s=2,c=32), BN, ReLU, MaxPool1D
ResNet Backbone	3 Blocks: [Conv1D × 2, BN, ReLU, Shortcut]
Channels	Layer 1: 32; Layer 2: 64; Layer 3: 128
Dual Pooling	GAP + GMP (concatenated, c=256)
Shared Neck	Linear (256→128), BN, ReLU, Dropout (0.5)
Embedding head	Linear (128→32), $\ell_{2}$-Normalization
Confidence head	Linear (128→64), BN, ReLU, Dropout (0.5), Linear (1)

*k*: kernel size; *s*: stride; *c*: output channels; BN: batch normalization; ReLU: rectified linear unit; GAP: global average pooling; GMP: global max pooling.

**Table 2:** Architectural specifications of the graph classifier.

Component	Specifications/Hyperparameters
Node Projector	Sinusoidal Encoding + Linear ($d_{in}\to 96$)
GNN Backbone	3 × [NNConv + LayerNorm]
Activation	LeakyReLU (α=0.1), Residual (ϵ-trainable)
Edge-MLP ($h_{\Theta }$)	Linear (3→64) → ReLU → Linear ($64\to 96^{2}$)
Global Readout	Attentional Aggregation
Classification Head	Linear (96→96), LeakyReLU, Dropout (0.5), Linear (96→3)

GNN: graph neural network; NNConv: edge-conditioned graph convolution operator; MLP: multilayer perceptron; LayerNorm: layer normalization; LeakyReLU: leaky rectified linear unit.

**Table 3:** Distribution of the topological dataset ($D_{T}$) by label complexity.

Group	PD Type	PD Tests	Avg. Pulses
	Corona	100	696
Clean Single-Source	Internal	100	687
	Surface	100	648
	Corona, Noise	100	7490
Noisy Single-Source	Internal, Noise	100	7382
	Surface, Noise	100	8238
	Corona, Internal, Noise	100	5830
Noisy Multi-Source	Corona, Surface, Noise	100	5324
	Internal, Surface, Noise	100	5401
**Total**		**900**	**4633**

**Table 4:** Composition of the cross-domain evaluation dataset ($D_{\mathrm{CD}}$) after preprocessing.

PD Type	Test Configuration	PD Tests	Avg. Pulses
Corona	Grounded needle	97	30
	Grounded plane	100	
Internal	In oil	50	114
	In solid	50	
Surface	Suspension insulator	50	1363
**Total**		**347**	**246**

**Table 5:** Hyperparameters of the graph construction stage.

Category	Parameter	Value
Node filtering	Confidence threshold ($\tau_{p}$)	0.5
	Minimum retained events	10
Edge construction	*k*-nearest neighbors (*k*)	5
	Morphological threshold ($\tau_{\mathrm{morph}}$)	0.3
	Morphological exponent (γ)	2.0

**Table 6:** Performance of the multi-task morphological network.

Head	Metric	Value
Embedding	Silhouette coefficient (*S*) ^a^	0.9121±0.0178
	NMI ^b^	1.0000±0.0000
Confidence	AUROC	0.9996±0.0003
	MCC (signal vs. noise) ^c^	0.9884±0.0046

^a^ Cosine Silhouette on $\ell_{2}$-normalized embeddings; ^b^ NMI via *k*-means with k=8 (physical sources in the test subset) averaged over ten restarts per seed; ^c^ evaluated at a threshold of $p_{i}=0.5$. Results reported on the test subset of $D_{M}$. Values are mean ± CI95 over five seeds.

**Table 7:** Overall multi-label classification performance on $D_{T}$.

Method	AUROC	MCC	EMR
Proposed (GNN)	1.00±0.00	0.98±0.02	0.97±0.03
Grid-CNN	0.92±0.04	0.72±0.08	0.69±0.09
Deep FNN	0.95±0.00	0.81±0.03	0.82±0.03
Linear SVM	0.94±0.00	0.78±0.01	0.76±0.01
Non-linear SVM	0.93±0.00	0.73±0.00	0.71±0.01
DeepSets	0.95±0.02	0.79±0.05	0.79±0.06
Set Transformer	0.88±0.03	0.64±0.05	0.67±0.06

Values reported as mean ± CI95 over five seeds, aggregated across all test conditions of the test subset of $D_{T}$. Bold values indicate the best result in each column.

**Table 8:** MCC by test condition on the test subset of $D_{T}$.

Method	Clean	Noisy	Multi
Proposed (GNN)	0.99±0.02	0.98±0.02	0.97±0.02
Grid-CNN	0.93±0.07	0.64±0.05	0.61±0.19
Deep FNN	1.00±0.00	0.83±0.04	0.60±0.05
Linear SVM	0.96±0.01	0.86±0.01	0.52±0.00
Non-linear SVM	1.00±0.00	0.77±0.01	0.44±0.00
DeepSets	0.92±0.04	0.84±0.09	0.62±0.08
Set Transformer	0.84±0.10	0.81±0.09	0.28±0.09

MCC reported as mean ± CI95 over five seeds (n=30 PD tests per condition). Bold values indicate the best result in each column. Clean: clean single-source; Noisy: single-source with noise; Multi: multi-source.

**Table 9:** Overall cross-domain classification performance on $D_{\mathrm{CD}}$.

Method	AUROC	MCC	EMR
Proposed (GNN)	1.00±0.00	0.93±0.04	0.94±0.03
Grid-CNN	0.38±0.06	−0.29±0.05	0.07±0.03
Deep FNN	0.88±0.03	0.61±0.07	0.67±0.08
Linear SVM	0.82±0.00	0.60±0.01	0.57±0.01
Non-linear SVM	0.72±0.00	−0.02±0.01	0.20±0.01
DeepSets	0.90±0.05	0.80±0.03	0.86±0.01
Set Transformer	1.00±0.01	0.89±0.09	0.93±0.06

Values reported as mean ± CI95 over five seeds, with all parameters frozen at their in-domain values (no retraining or fine-tuning). Evaluated on n=347 single-source PD tests. Bold values indicate the best result in each column.

**Table 10:** Per-class cross-domain MCC on $D_{\mathrm{CD}}$.

Method	Corona	Internal	Surface
Proposed (GNN)	0.94±0.06	0.87±0.06	0.99±0.02
Grid-CNN	0.00±0.00	0.00±0.22	−0.25±0.14
Deep FNN	0.71±0.05	0.51±0.07	0.37±0.10
Linear SVM	0.82±0.00	0.51±0.01	0.20±0.01
Non-linear SVM	0.53±0.00	−0.14±0.01	−0.23±0.02
DeepSets	1.00±0.01	0.74±0.04	0.16±0.28
Set Transformer	1.00±0.00	0.86±0.12	0.65±0.32

MCC reported as mean ± CI95 over five seeds. Bold values indicate the best result in each column. Per-class sample sizes: n=197 (Corona), 100 (Internal), 50 (Surface).

**Table 11:** Comparison of per-class grid-level sparsity between $D_{T}$ and $D_{\mathrm{CD}}$.

Dataset	Avg. Pulses	Non-Zero Bins (%)
$D_{T}$ — Corona	4093	19.1
$D_{T}$ — Internal	4035	18.8
$D_{T}$ — Surface	4443	14.7
$D_{\mathrm{CD}}$ — Corona	30	0.8
$D_{\mathrm{CD}}$ — Internal	114	2.8
$D_{\mathrm{CD}}$ — Surface	1363	11.0

**Table 12:** Hyperparameter sensitivity analysis on the topological dataset ($D_{T}$).

Hyperparameter	Value	MCC
	16	0.94±0.02
Embedding dimension (*d*)	**32**	0.98±0.02
	64	0.96±0.02
*k*-nearest neighbors (*k*)	3	0.95±0.02
	**5**	0.98±0.02
	7	0.97±0.02
	10	0.95±0.03
	0.3	0.93±0.03
Confidence threshold ($\tau_{p}$)	**0.5**	0.98±0.02
	0.7	0.96±0.02
Morph. threshold ($\tau_{\mathrm{morph}}$)	0.1	0.94±0.02
	**0.3**	0.98±0.02
	0.5	0.97±0.02
	0.7	0.92±0.03
	1.0	0.95±0.02
Morph. exponent (γ)	**2.0**	0.98±0.02
	3.0	0.97±0.02

Default values shown in bold. Results on the test subset of $D_{T}$.

**Table 13:** Component ablation on the contribution of morphological and confidence information.

Configuration	MCC
Full framework	0.98±0.02
Without morphological similarity ($W_{\mathrm{morph}}=1.0$)	0.89±0.03
Without confidence filtering ($p_{i}=1.0$ for all events)	0.84±0.04
Without both (morphological + confidence)	0.73±0.05

Results reported on the test subset of $D_{T}$. Bold indicates the best result.

**Table 14:** Stage I metrics under backbone architectural variants.

Configuration	S	NMI	MCC
Backbone depth (pooling fixed at GAP + GMP)			
2 blocks	0.85±0.03	0.95±0.02	0.97±0.01
3 blocks (default)	0.91±0.02	1.00±0.00	0.99±0.00
4 blocks	0.90±0.03	0.99±0.01	0.98±0.01
Pooling strategy (depth fixed at three blocks)			
GAP only	0.87±0.03	0.98±0.02	0.98±0.01
GMP only	0.86±0.03	0.97±0.02	0.97±0.01
GAP + GMP (default)	0.91±0.02	1.00±0.00	0.99±0.00

Values are mean ± CI95 over five seeds. Bold values indicate the best result in each column. *S*: Silhouette coefficient; NMI: normalized mutual information. The gating MCC is computed on the test subset of $D_{M}$.

**Table 15:** Graph topology by scenario on the test subset of $D_{T}$.

Scenario	Trials	Nodes *V*	Edges *E*	Density
Clean Single-Source	30	600±177	3001±885	0.009
Noisy Single-Source	30	430±124	2142±618	0.013
Multi-Source	30	715±159	3567±794	0.007
All (Test)	90	582±193	2903±966	0.010

Values reported as mean ± standard deviation across trials. Density: E/(V(V−1)), computed per trial and averaged.

**Table 16:** Per-method computational cost on a representative test trial (N=4622 pulses).

Method	Params	FLOPs (M)	Latency (ms)
Proposed (GNN)	2.0 M	60,676	116.9
Grid-CNN	102 K	68	37.1
Deep FNN	2.0 M	4	36.9
DeepSets	1.2 M	45,912	64.5
Set Transformer	546 K	45,267	68.4

Latency is the median over multiple repetitions on the same input. Proposed breakdown: Stage I 63.9 ms; Stage II 43.4 ms; Stage III 9.6 ms. Grid-CNN/Deep FNN: PRPD rendering 36.5 ms; model forward ≤1 ms. DeepSets/Set Transformer: Stage I 63.9 ms; set aggregator 0.6 ms and 4.4 ms, respectively. The two SVM baselines are omitted, as their kernel inference is not directly comparable in floating-point operations.

**Table 17:** Inference latency as a function of input size *N*.

*N*	$N_{f}$	*E*	St. I	St. II	St. III	Total
100	3	2	3.1	0.4	3.0	7.2
200	7	22	3.7	0.6	3.0	7.4
500	18	90	6.9	1.2	2.9	11.0
1000	39	195	13.8	2.0	3.0	18.9
2000	76	380	27.1	3.5	2.9	33.4
5000	196	980	67.5	8.5	3.4	79.8
10,000	371	1855	136.3	16.4	5.1	158.1

Timings in milliseconds, median over repetitions per *N*. $N_{f}$: retained nodes; *E*: retained edges. St. I: encoder; St. II: graph build; St. III: GNN. Each column reports an independently measured median, and the total is the median of the directly measured end-to-end latency rather than the sum of the per-stage medians. Because the median of a sum is not in general the sum of the medians and the stages incur inter-stage overhead, the per-stage columns need not add up exactly to the total.

## Data Availability

The cross-domain evaluation dataset analyzed in this study is publicly available at IEEE Dataport (https://dx.doi.org/10.21227/a3r5-zs61) [33]. The Smart Grids Laboratory (LRI) data used for training, validation, and testing are not publicly available due to ongoing research and confidentiality agreements with the funding partner; reasonable requests for academic access may be directed to the corresponding author. The source code implementing the proposed framework together with the trained model weights and configuration files required to reproduce the reported results is available from the corresponding author upon reasonable request.

## Abbreviations

The following abbreviations are used in this manuscript:

AC	Alternating current
AUROC	Area under the receiver operating characteristic curve
BN	Batch normalization
CBM	Condition-based maintenance
CI95	95% confidence interval
CNN	Convolutional neural network
Edge-MLP	Edge-conditioned multilayer perceptron
EMR	Exact match ratio
FLOPs	Floating-point operations
FNN	Feedforward neural network
GAP	Global average pooling
GIS	Gas-insulated switchgear
GMP	Global max pooling
GNN	Graph neural network
HFCT	High-frequency current transformer

ISAB	Induced set attention block
LRI	Smart Grids Laboratory (*Laboratório de Redes Inteligentes*)
MCC	Matthews correlation coefficient
MLP	Multilayer perceptron
NMI	Normalized mutual information
NNConv	Edge-conditioned graph convolution operator
PD	Partial discharge
PRPD	Phase-resolved partial discharge
RBF	Radial basis function
ResNet	Residual network
SVM	Support vector machine
t-SNE	t-distributed stochastic neighbor embedding
XLPE	Cross-linked polyethylene
